# Measurement of Epicardial Fat Thickness by Echocardiography Presents Challenges

**DOI:** 10.5935/abc.20160167

**Published:** 2016-11

**Authors:** Mustafa Gulgun, Fatih Alparslan Genç

**Affiliations:** Gulhane Education and Research Hospital, Pediatric Cardiology, Turkey

**Keywords:** Pericardium, Adipose Tissue, Electrocardiography, Arrhythmias, Cardiac, Echocardiography, Reference Values, Magnetic Resonance Imaging / trends

**To the Editor,**

It was with great interest that we read the paper by Kaplan et al.^1^ entitled ''Evaluation of Electrocardiographic T-peak to T-end Interval in Subjects with Increased Epicardial Fat Tissue Thickness'' published on *Arquivos Brasileiros de Cardiologia* in December 2015. They aimed to investigate the relationship between epicardial adipose tissue (EFT) and ventricular repolarization. They demonstrated some ventricular repolarization abnormalities on electrocardiography in patients with higher EFT thickness. We would like to thank Kaplan et al.^1^ for their great effort in this study, and share some of our thoughts.

Echocardiographic measurement of EFT is a commonly used imaging modality because it presents advantageous characteristics such as easiness, reproductibility, low-cost, availability and lack of radiation. However, it presents technical difficulties which may have an effect on the interpretation of study results.^2^ First, two-dimensional echocardiography gives us a linear measurement of EFT. Since EFT is a three-dimentional structure, two-dimensional echocardiography can only partially measure the thickness of EFT and can not give us precise volume of EFT. Second, obtaining clear acoustic windows with echocardiography is not easy in obese individuals. Also, the poor intraobserver and interobserver variability compared to cardiac magnetic resonance imaging or computerized tomography is still an issue. Moreover, an inattentive sonographer can measure the pericardial effusion or pericardial adipose tissue thickness instead of EFT.^2^ Finally, EFT can change with supine or lateral positioning during echocardiography.^3^ In the study by Kaplan et al.,^1^ the measurement of EFT was performed perpendicularly on the free wall of the right ventricle at the end diastole in the parasternal long-axis view in three cardiac cycles. We believe the measurement can be performed in the parasternal short-axis view, as well as parasternal long-axis view, and the mean value of the measurements may be calculated subsequently. Thus, one can maximize the percentage of accurate measurement for EFT.^2,3^ In addition, the measurements performed at the end-systole and end-diastole at the same time could have made this study more valuable for future researchers.^4^

Today, magnetic resonance imaging is the gold standard for visceral adipose tissue assessment and computerized tomography can measure the EFT and volume more accurately than echocardiography.^5^ It would have been very helpful to perform magnetic resonance imaging for the evaluation of EFT in terms of defining the exact role of EFT thickness in ventricular repolarization abnormalities which has prognostic importance.
