# Supplementary material for: Measurement of Epicardial Fat Thickness by Echocardiography Presents Challenges
Source: Arq Bras Cardiol. 2016 Nov;107(5):497–8. doi: 10.5935/abc.20160167 (PMC5137397; doi:10.5935/abc.20160167)
Supplement: Related texts

# Reply

**Authors:** Özgür Kaplan, Ertugrul Kurtoglu

We thank the authors for their interest in our article. Firstly, we agree, in part, with the authors' assertion that the two-dimensional echocardiography can only partially measure the thickness of epicardial fat tissue (EFT) and cannot give us the precise volume of EFT. However, three-dimensinal echocardiographic evaluation of EFT is time-consuming and is not a cost-effective method. Therefore, we used a simple, ready to use, and reproducible method as described by Iacobellis et al.^1^ In addition, in our study, long and short axis measurements were calculated, but clear and reliable acoustic windows were observed in the long axis views. When we compared long and short axis views in our study, we found that EFT measurements were similiar. Therefore, we used long axis views. These findings are in line with the findings by a previous study, which found that both short and long axis measurements were similiar.^2,3^ Secondly, the authors state that obtaining clear acoustic windows with echocardiography is not easy in obese individuals. Every clinician knows that obese individuals are not good candidates for two or three-dimensional echocardiographic evaluations. However, our patients were overweight and not obese, since the mean BMI in our study was 28 kg/m^2^. Any participant who did not present a clear acoustic window was excluded from the study participation as cited in Methods section. Thirdly, the authors say that the poor intraobserver and interobserver variability compared to cardiac magnetic resonance imaging (MRI) or computerized tomography (CT) is still an issue. We did not utilize MRI or CT in our study, and intra and inter-observer variablity may be an issue with an study. Finally, the authors state that an inattentive sonographer can measure the pericardial effusion instead of EFT, and EFT can change with supine or lateral positioning during echocardiography. The sonographer in our study was a cardiologist who had expertised in echocardiography. Also, stardard EFT evaluation for echocardiography is parasternal axis in left lateral decubitis position; and for CT or MRI, it is supine position. Therefore, it does not matter that EFT can change with supine or lateral positioning as long as a standardized method is used for any measurement by any method.

**Özgür Kaplan****Ertugrul Kurtoglu**

## References

[1] Iacobellis G, Ribaudo MC, Assael F, Vecci E, Tiberti C, Zappaterreno A (2003). Echocardiographic epicardial adipose tissue is related to
anthropometric and clinical parameters of metabolic syndrome: a new
indicator of cardiovascular risk. J Clin Endocrinol Metab.

[2] Eroglu S, Sade LE, Yildirir A, Demir O, Müderrisoglu H (2013). Association of epicardial adipose tissue thickness by
echocardiography and hypertension. Turk Kardiyol Dern Ars.

[3] Eroglu S, Sade LE, Yildirir A, Bal U, Ozbicer S, Ozgul AS (2009). Epicardial adipose tissue thickness by echocardiography is a
marker for the presence and severity of coronary artery
disease. Nutr Metab Cardiovasc Dis.
